# Low temperature upregulates *cwp* expression and modifies alternative splicing patterns, increasing the severity of *cwp*-induced tomato fruit cuticular microfissures

**DOI:** 10.1038/s41438-019-0204-9

**Published:** 2019-11-08

**Authors:** Noam Chechanovsky, Ran Hovav, Rina Frenkel, Adi Faigenboim, Yelena Eselson, Marina Petreikov, Michal Moy, Shmuel Shen, Arthur A. Schaffer

**Affiliations:** Department of Vegetable and Field Crops, Institute of Plant Sciences, Agricultural Research Organization, Rishon LeZion, Israel

**Keywords:** Plant molecular biology, Gene regulation

## Abstract

The *cwp* (cuticular water permeability) gene controls the development of cuticular microfissuring and subsequent fruit dehydration in tomato. The gene underwent silencing in the evolution of the fleshy cultivated tomato but is expressed in the primitive wild tomato relatives. The introgression of the expressed allele from the wild *S. habrochaites* (*cwp*^*h*^) into the cultivated tomato (*Solanum lycopersicum*) leads to the phenotype of fruit water loss during and following ripening. In this report, we show that low temperature impacts on the severity of the cuticular microfissure phenotype via a combination of effects on both expression and alternative splicing of *cwp*^*h*^. The *cwp* gene, comprising four exons and three introns, undergoes post-transcriptional alternative splicing processes, leading to seven alternative transcripts that differ in reading-frame lengths. Transgenic plants expressing each of the alternative transcripts identified the longest reading frame (VAR1) as the functional splice variant. Low temperature led to a strong upregulation of *cwp*^*h*^ expression, compounded by an increase in the relative proportion of the functional VAR1 transcript, leading to increased severity of microfissuring of the cuticle. In summary, we demonstrate the molecular mechanism behind the horticultural phenomenon of the low-temperature effect on cuticular microfissures in the dehydrating tomato.

## Introduction

The aerial organs of all plants are covered by a thin layer composed of a polyester matrix of cutin and organic waxes, the cuticle, in concert with the epidermal cell walls, which protects the inner plant organs against biotic and abiotic stresses, especially loss of water^1,2^. The cultivated tomato fruit is covered by a relatively thick cuticle and lacks stomata, making the fleshy fruit cuticle a continuous tissue relatively impervious to water loss even following harvest^3–5^. The fruits of the various wild tomato relatives exhibit a wide range in size, color, and texture^6^, including diverse cuticular architecture and chemical composition, but the relatively thick cuticle is particularly characteristic of the cultivated *S. lycopersicon*^7^.

Fleshy fruit may develop fissures in the cuticle, of varying sizes and spatial organization, and this is true as well for tomato^8^. Cuticular microfissures are generally considered an undesirable phenotype and can lead to reduction in fruit quality and yield loss^8,9^. In tomato fruit, they are controlled both by genetic factors, including genes that regulate the biosynthesis of the cuticle and cell wall components^10–15^ and by environmental factors, including irrigation, temperature, humidity, and intense exposure to radiation^8,16,17^. The resultant chemical components and the related physical and chemical structure impacts on the biomechanical characteristics of the fruit cuticle tissue^18–21^.

Previously, we identified the gene *cwp* (*c*uticular *w*ater *p*ermeability) whose expression underwent silencing during the evolution and domestication of the cultivated fleshy fruit tomato^3^. While the allele from the cultivated *S. lycopersicon* (*cwp*^*e*^, from the former nomenclature *Lycoperiscum esculentum*) is not expressed, the alleles of the primitive green-fruited species are expressed during early fruit development. Significantly, when the *cwp* gene is expressed in the cultivated tomato background, either by introgression of the *cwp* allele from primitive wild tomato species, such as *Solanum habrochaites* (*cwp*^*h*^), or by transgenic overexpression, microfissures develop in the fruit cuticle, leading to the subsequent dehydration of the fruit. The importance of *cwp* is suggested by its ubiquitous expression in other members of the *Solanaceae*, e.g., tobacco (*Nicotiana tabacum*), pepper (*Capsicum annuum*), potato (*Solanum tuberosum*), and the green-fruited tomato wild relatives of tomato;^3^ however, the function of *cwp* is, yet, undetermined.

Over the years, horticultural growers of the dehydrating tomato have taken note that the severity of microfissures in fruit of *cwp*^*h*^ introgression lines was more pronounced when plants were grown in the greenhouse during the fall–winter (generally minimum night temperature of 15 °C) than when grown in spring–summer with higher day and night temperatures. This led us to study the role of low temperature on *cwp*^*h*^-induced microfissuring, hypothesizing that there is a molecular–genetic component in the temperature effect on *cwp*^*h*^.

It is well established that temperature changes affect gene expression in plants in general, and in tomatoes as well, leading to differential up- and downregulation of temperature-responsive genes^22–25^. In addition to the regulation of gene transcription, the role of temperature on alternative splicing of gene transcripts is also well established^25–28^, and the involvement of alternative splicing in response to abiotic stress has been the topic of exhaustive reviews^29–32^. Alternative splicing generates multiple mRNA and protein isoforms from a single gene and is common in all eukaryotes^33,34^. It was initially reported in plants nearly 30 years ago^35^ following earlier reports in animals. In plants, a significant number of intron-containing genes undergo alternative splicing process. More than half the intron-containing genes of Arabidopsis are subject to alternative splicing^36–38^, and a survey of nine species^39^ indicated that between 40 and 70% of the expressed intron-containing genes underwent alternative splicing. In an early study of tomato, Aoki et al.^40^ used cDNA library shotgun sequencing and found that only about 10% of the genes undergo alternative splicing, but more recently, Sun and Xiao^27^ by using HTS methods, showed that ca. 60% of the genes expressed in young tomato fruit exhibited alternative splicing. Most of alternative splicing events in plants are intron-retention type (61–68%), followed by alternative 3′ and alternative 5′ (25–28% together) and the rarest event of exon skipping (2–5%)^41^.

In this report, we show that the effect of low temperature on cuticular microfissuring and dehydration in *cwp*-expressing tomato fruit is via both the upregulation of *cwp*, compounded by alternative splicing of the *cwp* gene.

## Materials and methods

### Plant material

The segregating introgression line (SIL) for *cwp*^*h*^ and *cwp*^*e*^ used in this study is as described in Hovav et al.^3^. The origin of the population is a backcross of the wild tomato species *S. habrochaites* LA1777 to the cultivated tomato (*S. lycopersicum*). A second segregating line, of miniature-sized plants for study in growth chambers, was developed from a BC3F2 of the *cwp*^*h*^ SIL to *S. lycopersicum* var. Microtom^42^ accompanied by genotyping for the *cwp*^*h*^ and *cwp*^*e*^ alleles based on polymorphisms by using primers CWP-F 5′-CGTACTCAAACGATGATAAAGGT-3′ and CWP-R 5′-TTATTGCATTTGGAGTTTTTCAATCCG-3′.

#### Temperature treatments for whole plants

Plants were grown under standard conditions, as described previously^43^. For the growth of the Microtom-type (MT) plants under controlled temperature conditions, six plants of each of the *cwp* genotypes were grown in 500-ml pots until flowering. The flowering plants were transferred into two controlled growth chambers (Percival) set to temperatures 20/30 °C (night/day, high temperature) and 10/15 °C (night/day, low temperature). Flowers were marked at anthesis at the beginning of the temperature treatments. Following 21 days of incubation, five IG fruits from each plant were used for RNA extraction for *cwp* expression-level measurement, and the plants were transferred to a greenhouse for further development. Ripe fruits were picked from all plants, and the microfissure level was phenotyped as described below.

#### Temperature treatments for detached fruit

Temperature treatments were applied to detached IG fruit of either the SIL or the MT plants by placing the fruits under humid conditions in temperature-controlled chambers at temperatures (4, 10, 20, and 30 °C) and length of period (10 or 72 h), as described in this paper.

### *CWP* expression level by quantitative PCR

The expression level of *cwp* was measured by quantitative PCR (qPCR) by using Maxima^®^ SYBR-Green (Fermentas). In brief, first-strand cDNA was synthesized on template RNA that was extracted from tomato fruit pericarp. A qPCR reaction was conducted on 1 μl of cDNA as a template, with the primers qCWP-F 5′-CTATTTTCGTGGAAGTTGACAC-3′ and qCWP-R 5′-TGACAATTTGCTCTTTCCAC-3′ for amplification of *CWP*. The primers q18S-F 5′-GTGCATGGCCGTTCTTAGTTG-3′ and q18S-R 5′-CAGGCTGAGGTCTCGTTCGT-3′ were used for amplification of 18S rRNA as control. The relative expression level of *cwp* was calculated as the ratio of absolute expression of *cwp* to the absolute expression of 18S rRNA, by the equation

$$CWP_R = \left( {CWP_{abs}/18S_{abs}} \right)\, \times \,1000$$

where *CWP*_*R*_ is the relative expression level of *CWP*, *CWP*_*abs*_ is the absolute expression level of *CWP*, and *18S*_*abs*_ is the absolute expression level of 18S rRNA.

### RNAseq of near-isogenic SIL fruit

For the study of global gene expression during fruit development, the SIL line segregants were grown, and fruit pericarp and peel were sampled from immature green (IG), mature green (MG), and breaker (Br) stages. For the study of the effect of temperature on detached fruitlets, IG fruitlets of MT BC_3_F_7_ lines were sampled and kept for 10 h, either at 20 or at 4 °C. Two replications, each of a minimum of three fruits, were prepared for each MT genotype. Tissues containing the skin and outer pericarp were sampled and immediately frozen in liquid nitrogen. Samples were prepared for RNAseq and run on Illumina HiSeq2000, as described in Shammai et al.^44^.

### Bioinformatics—statistics and programs

#### Statistics of RNAseq

Raw reads were subjected to a filtering and cleaning procedure. The SortMeRNA tool^45^ was used to filter out rRNA, and the FASTX Toolkit (http://hannonlab.cshl.edu/fastx_toolkit/index.html, version 0.0.13.2) was used to trim read-end nucleotides with quality scores <30, by using the FASTQ Quality Trimmer, and to remove reads with <70% base pairs with a quality score ≤30 by using the FASTQ Quality Filter. Statistics of the reads and quality are presented in Supplementary Table 1.

#### Mapping and expression analysis

Clean reads were aligned to the tomato genome extracted from the Sol Genomic Network database (https://solgenomics.net/organism/Solanum_lycopersicum/genome; SL3.0) by using Tophat2 software (v2.1^46^). Gene abundance estimation was performed by using Cufflinks (v2.2^47^) combined with gene annotations (ITAG3.10). Differential expression analysis was completed by using the edgeR R package^48^. Genes that varied more than twofold, with an adjusted *p* value of no more than 0.05, were considered differentially expressed. Venn diagrams were generated by using the online tool at bioinformatics.psb.ugent.be/webtools/Venn/. Functional annotation of the significantly expressed genes was prepared by using Plant MetGenMAP tool (http://bioinfo.bti.cornell.edu/cgi-bin/MetGenMAP/home.cgi). Heatmap visualization was performed by using R Bioconductor^49^.

#### Alternative splicing analysis

Intron abundance estimation was performed by using htseq-count (https://htseq.readthedocs.io/en/release_0.11.1/count.html) combined with gene annotations (ITAG3.10). Differential intron expression analysis was completed by using the edgeR R package.

#### Splice-related gene expression

Tomato splice-related genes were identified by using homology search (BLAST) against the Arabidopsis Splicing Related Gene Database (SRGD, http://www.plantgdb.org/SRGD/WB04.php, Wang and Brendel^50^).

### Cloning the alternative transcripts of *cwp*

Total RNA from young tomato fruits of genotype *cwp*^*h*^ was extracted by using the Tri-Reagent (Invitrogene) according to the manufacturer’s instructions. First-strand cDNA was synthesized from the RNA template by using AMV reverse-transcriptase enzyme (Fermentas). The coding sequence (CDS) of the alternative transcripts was amplified by PCR reaction conducted on the cDNA, by using the primers CWP5UTR-F 5′-TCTTCATCTTATTCTTGTTTTTATTTATAG-3′ and the primer CWP3END-R 5′-TTATTGCATTTGGAGTTTTTCAATCCG-3′. The amplified fragments, comprising the seven alternative *cwp* transcripts, were cloned into pGEM^®^-Teasy plasmid (Promega) according to the manufacturer’s protocol. The plasmids were introduced into *E. coli* strain JM109 (Promega) by heat-shock treatment, and the bacteria planted on LB-agar plates containing 100 mg/l ampicillin and x-gal as selection markers. White colonies were subjected to PCR by using the universal primers SP6 and T7. Plasmids containing the sequences of the seven alternative variants were named pGEMAS1–pGEMAS7 (alternative transcripts VAR1– VAR7; correspondingly, sequences are presented in Supplementary File 1) and kept at −20 °C until further use.

### Transgenic plants for the seven alternative transcripts

The coding sequence of the alternative transcripts was amplified by PCR reaction performed on the plasmids pGEMAS1– pGEMAS7, by using the primers CWPASNcoI-F that includes a restriction site for the restriction enzyme (RE) NcoI 5′-CATCCCATGGGTATAGTAGTGTTTATTTGGG-3′ (NcoI site is underlined), and CWPASSalI-R that includes a restriction site for the RE SalI 5′-CATGTCGACTTATTGCATTTGGAGTTTTTCAATCCG-3′ (SalI site is underlined). The PCR products were further cloned by using the pART7/pART27 binary system^51^. The resultant pART27 plasmids, under the control of 35S promoter, including the *NptII* gene (for kanamycin resistance) as marker selection, were introduced into *Agrobacterium tumefaciens* by using electroporation. Transgenic tomato plants (var. MP-1) were produced by the *Agrobacterium* method as described in ref. ^3^. Successful transformant plants were selected by PCR reaction conducted on the marker selection *NptII*, by using the primers NPTII-F 5′-TGAATGAACTGCAGGACGAG-3′ and NPTII-R 5′-AGCCAACGTATGTCCTGAT-3′. At least five independent T_0_ plants were selected for each of the alternative spliced coding sequences, and the selected plants were grown in a greenhouse and allowed to self-pollinate to produce T_1_ populations. T_1_ plants were grown as above, and mature green and ripe fruits were scored for microfissures and dehydration.

### Quantitative measurement of alternative transcripts

Total RNA was extracted from detached IG tomato fruits of the SIL population, following incubation at three temperatures (10 , 20, and 30 °C) for 72 h. The cDNA was amplified by PCR by using the primers CWP5UTR-F and CWP3END-R as described above. PCR products were cloned to pGEM-Teasy vector, and the plasmids were introduced into *E. coli* strain JM109 by heat shock as described above. After transformation, bacteria were grown on agar plates containing 100 mg/l ampicillin and x-gal. At least 100 white colonies, each colony comprising a single transcription variant, were selected randomly for PCR reaction by using the universal primers SP6 and T7. The PCR products were separated on 2% agarose gel, together with the PCR products of the pGEMAS plasmids as size markers for the seven alternative transcripts. Number of each alternative transcript, measured by comparing its size to the size markers, was calculated based on the total alternative transcripts in three replications from each temperature treatment.

## Results

Our previous results indicated that *cwp* is expressed in immature fruit of the green-fruited primitive wild species of tomato, including *S. habrochaites*, and in introgression lines derived from it harboring the *habrochaites* Solyc04g082540 ortholog, *cwp*^*h*^. In contrast, the *S. lycopersicon cwp*^*e*^ allele is not expressed. In this study, RNAseq results of developing tomato fruit of the segregating SIL corroborated this, showing that the *cwp*^*h*^ allele is expressed only transiently in the IG stage in fruit pericarp, while the *cwp*^*e*^ allele is not expressed during development (Table 1).

**Table 1:** Effect of *cwp* genotype on *cwp* gene expression in segregating introgression lines (SIL population)

Fruit stage	FPKM values for Solyc04g082540	
	*cwp* ^*h*^	*cwp* ^*e*^
IG	58	<1
MG	<1	<1
Br	<1	<1

Results are FPKM values for Solyc04g082540 determined from RNAseq analysis of developing tomato fruit pericarp. The data represent two replications each of a minimum of three fruits. *cwp*^*h*^ indicates the wild species allele from *S. habrochaites* and *cwp*^*e*^ indicates the cultivated tomato allele

### The effect of temperature on cuticular microfissures

**Fig. 1:**
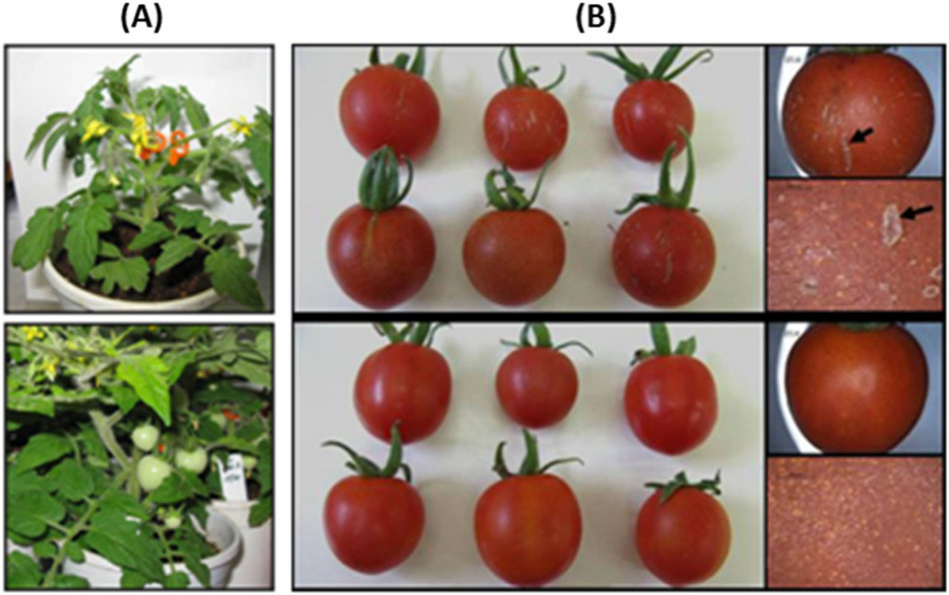
Effect of temperature at IG stage on microfissuring of mature fruit. Plants of genotype *cwp*^*h*^ in the MT background were grown in two ambient temperature ranges: low (10/15 °C night/day) and high (20/30 °C night/day) and tested for microcracking phenotype on the fruit cuticle. **a** Flowers were marked prior to the 2-week incubation (top), and the fruits continued to develop after the differential temperature incubation (bottom) in the greenhouse. **b** Representative ripe fruit from the temperature treatments (top, low temperature; bottom, high temperature). Incubation in low temperatures led to severer phenotype and deep cuticular “scars” (**b**, right top, arrow points to suberized scar) compared with high temperatures (**b**, right bottom)

We observed over a number of seasons that the microfissure phenotype in cultivated tomato fruits of genotype *cwp*^*h*^ is generally more severe in winter-grown crops than in summer-grown crops. In the warmer weather, the fruits exhibit delicate and more sporadic microfissures, in comparison with winter. In order to study the effect of temperature during the early fruit development stage on microfissures, we developed miniature tomato plants of *cwp*^*h*^ and *cwp*^*e*^ by backcrossing the cultivated tomato *cwp*^*h*^-SIL to var. Microtom for three generations, selfing the heterozygous *cwp*^*he*^, and then selecting for the *cwp* allele genotypes by PCR. Six flowering plants of each genotype were grown in growth chambers for 3 weeks at low temperature (10/15 °C night/day) or high temperature (20/30 °C night/day) and then returned to a common greenhouse for continued development. Although both temperature conditions led to the microfissured cuticle on all fruits, significant differences were observed in the intensity of the microfissures. In response to low temperature, the fruits exhibited dense microfissures with deep scars, accompanied by suberization, while under the warm-temperature treatment, the fruits exhibited reduced microfissures and no scars were observed (Fig. 1).

### Expression of the *cwp*^*h*^ allele is upregulated under low temperature

**Fig. 2:**
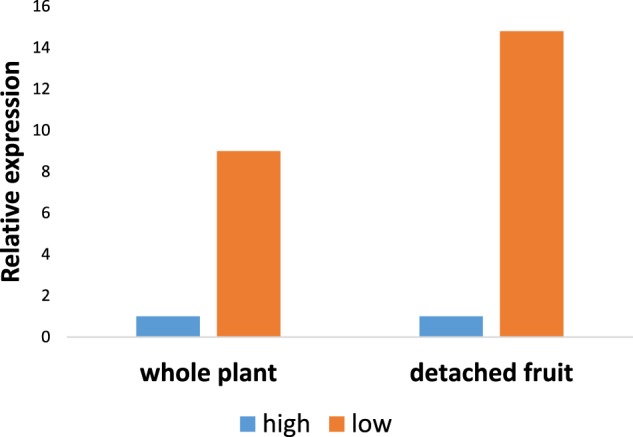
Expression level of *cwp*^*h*^ after incubation in two temperatures, low (10/15 °C) and high (20/30 °C). The left portion of the graph represents the expression level in IG fruitlets of whole plants following 21 days of temperature treatment. The right side of the graph indicates the expression of detached IG fruitlets treated for 72 h. Expression values are presented as relative to the high-temperature treatment in each experiment. Expression of *cwp*^*e*^ fruitlets was nil

We measured the expression of *cwp* in the IG fruit at the end of the 3-week temperature treatment and found that it was strongly upregulated by low temperature by about tenfold (Fig. 2a). In order to test whether the effect of low temperature on *cwp* expression is sensed by the fruit itself, rather than by the whole plant, we detached IG fruit of the two genotypes and maintained them at the same temperatures as above, for 72 h. The results showed that *cwp*^*h*^ gene expression in the detached fruit was increased about 15-fold in response to low temperature, similar to the whole plant (Fig. 2b). The silence of *cwp*^*e*^ expression was not affected by the low temperature (not shown).

### Global gene expression of developing fruit is affected by temperature

In order to further study the effect of low temperature on IG fruit gene expression, we performed a RNAseq study comparing global gene expression in detached IG fruits of the two *cwp* genotypes in the MT background, kept for 10 h at either 20 or 4 °C. The results further corroborated the upregulation of *cwp* gene expression in response to low temperature and further extended the temperature effect to the short time period of overnight (Table 2). Again, only the *cwp*^*h*^ allele is upregulated in response to low temperature; the *cwp*^*e*^ allele remained silent and was not affected by low temperature.

**Table 2:** Effect of 10-h temperature treatment on *cwp* gene expression in detached IG fruit from MT introgression lines

*cwp* Genotype	Temperature treatment	
	20 °C	4 °C
*cwp* ^*e*^	<1	<1
*cwp* ^*h*^	62	132

Results are FPKM values for Solyc04g082540 determined from RNAseq analysis. The data represent two replications each of a minimum of three fruits

**Fig. 3:**
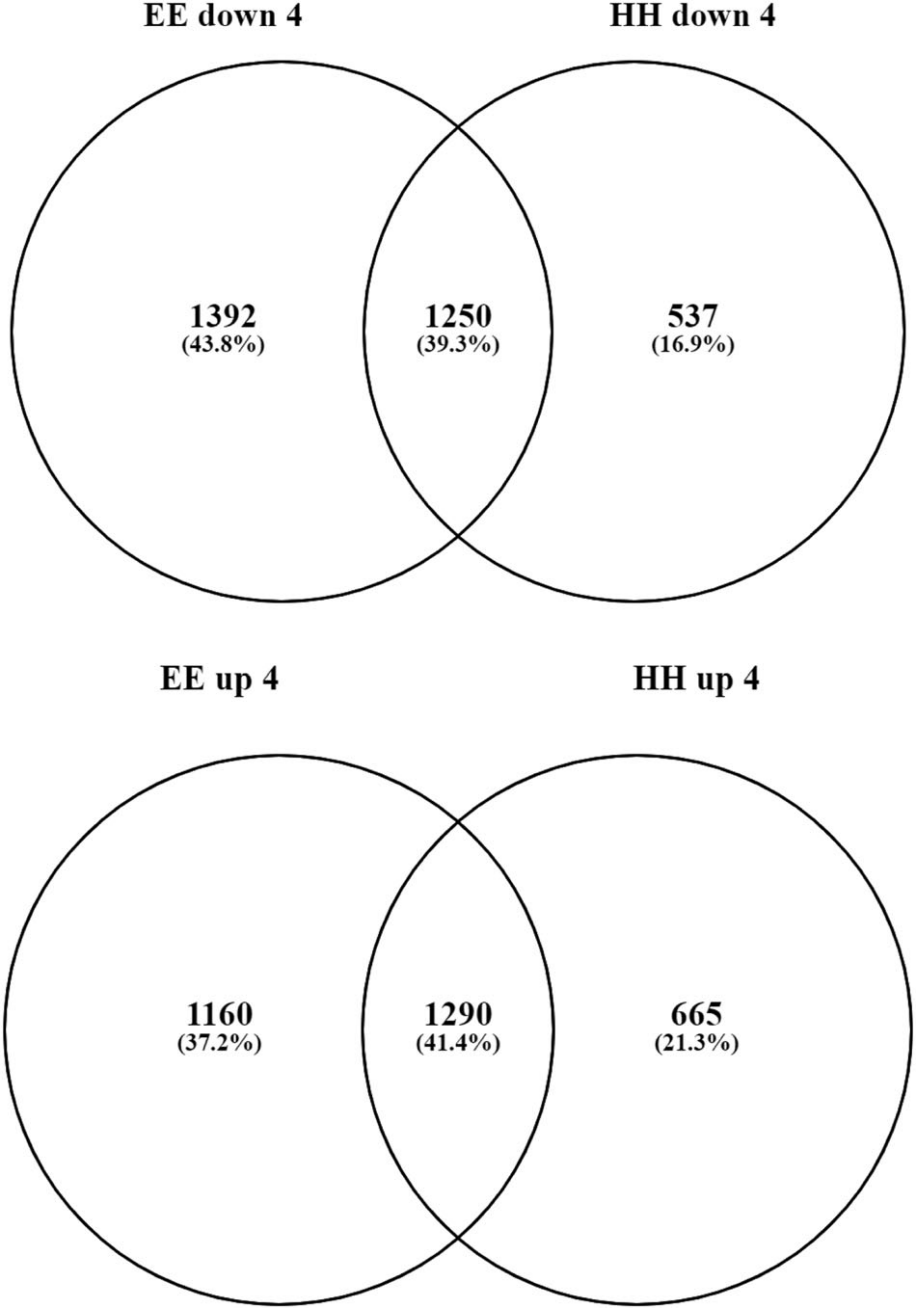
Differential gene expression due to low temperature. Venn diagrams indicating the overlap between the two *cwp* genotypes for the differentially downregulated (upper diagram) and upregulated (lower diagram) genes in response to 4 °C treatment

The *cwp*^*h*^ gene is not unique with regard to being strongly regulated in response to low temperature. Whole-transcriptome analysis indicated significant differential regulation of transcription, and both genotypes showed temperature-dependent regulation. Fruit of the *cwp*^*e*^ genotype had 2350 genes upregulated and 2642 genes downregulated by the low temperature, while the *cwp*^*h*^ genotype exhibited 3741 differentially regulated genes (1955 upregulated and 1786 downregulated), by at least twofold by low temperature. Common to both genotypes were 1290 upregulated, and 1249 downregulated, genes (Fig. 3, Supplementary Excel File 1). Between the two genotypes, a total of ca. 6000 genes were differentially expressed in response to temperature, comprising ca. 25% of the expressed genome.

### *cwp*^*h*^ undergoes alternative splicing and produces seven alternative variants

**Fig. 4:**
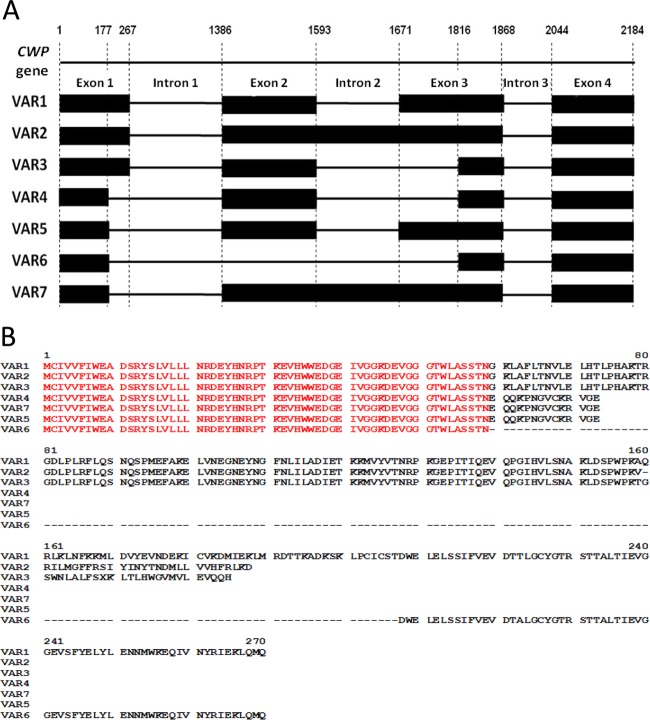
A schematic view of the seven *cwp* alternative splicing variants. **a** Genomic DNA alignments indicating the splicing variants. Wide black lines represent exons, narrow black lines represent introns. **b** Clustal multiple alignment of the amino acids of the proteins of the seven alternative transcripts

PCR of the *cwp*^*h*^ cDNA indicated multiple alternative transcripts (Supplementary Fig. 1a). Sequencing of the multiple PCR products indicated seven alternative transcripts of the *cwp* gene, termed VAR1–VAR7 (Fig. 4). VAR1 was the longest sequence, the product of the removal of three introns and retention of four exons. In comparison with VAR1, the other variants are a result of four different alternative splicing events: intron 2 retention, alternative 3′ acceptor of intron 2, alternative 5′ donor of intron 1, and exon 2 skipping. VAR2 undergoes an intron 2 retention, VAR3 is processed via alternative 3′ acceptor of intron 2, VAR4 results from a combination of alternative 3′ acceptor of intron 2 and alternative 5′ donor of intron 1, VAR5 undergoes alternative 5′ donor of intron 1, VAR6 is formed by a combination of alternative 3′ acceptor of intron 2, alternative 5′ donor of intron 1, and exon 2 skipping, and VAR7 is formed by a combination of intron 2 retention and alternative 5′ donor of intron 1. All the introns of the *cwp*^*h*^ alternative splicing variants are U2 type, and follow all the U2-type intron rules: (1) the sequence AG/GUAAGU (exon/intron, underlined are the conserved bases GU) at the 5′ junction of the intron, (2) the sequence UGYAG/GU (intron/exon, underlined are the conserved bases AG) at the 3′ junction of the intron, (3) U/A-rich regions, (4) a conserved YURAY sequence in the branchpoint, 30–70 bp upstream to the 3′ junction of the intron, and (5) a poly-U tract between the YURAY sequence and the 3′ junction of the intron (Supplementary Fig. 2).

Interestingly, when *cwp*^*h*^ cDNA from wild species *S. habrochaites* fruit, as well as from other primitive species that express the *cwp* gene, was analyzed for alternative transcripts, only three were observed, compared with the seven produced in the *S. lycopersicon* background of the introgression line (Supplementary Fig. 1b). This suggests that the pre-mRNA of *cwp*^*h*^ may be differentially spliced, depending on the endogenous splicing machinery, which likely differs between the wild species and the cultivated species.

### The seven alternative transcripts comprise reading frames for five proteins

The reading frames of the alternative transcripts of *cwp*^*h*^ differ significantly in length (Fig. 4, Supplementary Fig. 2, Supplementary Table 2). The longest reading frame was found in VAR1 with 811 bp in length coding for a 270 aa protein. VAR2 is the longest variant with 889 bp but a reading frame for a shortened protein of 187 aa, due to a premature stop codon in exon 3. The length of VAR3 is 666 bp with a reading frame for a 185-bp protein, also due to a premature stop codon in exon 3. VAR4, VAR5, and VAR7 share the same reading frame of 73 aa due to a premature stop codon in exon 2. VAR6 is dramatically shorter than the other variants due to its unique exon 2 skipping, but its reading frame encodes for a 122 aa protein. In total, five different potential proteins were identified, products of the seven alternative transcripts. All five proteins share a 73 aa N terminus, while the three longest proteins (products of VAR1, VAR2, and VAR3) share a longer region of 158 aa at the N terminus. VAR6 contains the 73 aa at the N terminus, and shares the C terminus with VAR1, due to the reversion to the reading frame (Fig. 4b).

### Functional analysis of alternative VARs

**Fig. 5:**
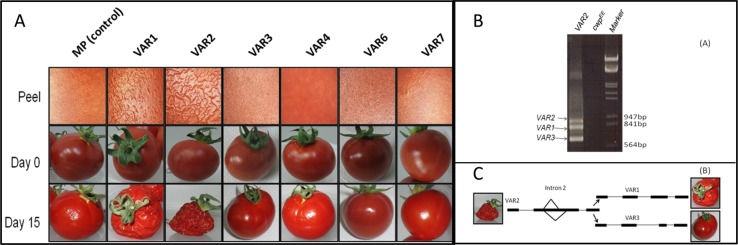
Ripe fruits of transgenic tomatoes for the *cwp* alternative splicing variants. **a** Microfissures were observed on the fruit peel of variants VAR1 and VAR2 only (upper row), and VAR1 and VAR2 were the only variants exhibiting dehydration after 15 days. **b** VAR2 itself undergoes alternative splicing, and PCR on VAR2 transgenic tomato results in three bands, VAR1, VAR2, and VAR3. **c** A schematic illustration of the alternative splicing events in intron 2 that leads to the production of VAR1 and VAR3 from VAR2

In order to determine which of the different splice variants are functional, we developed transgenic tomato plants for each (except VAR5 that shares the same reading frame as VAR4 and VAR7) in the background of variety MP-1, which we previously used to functionally express the *cwp* gene^3^. The results clearly showed that only transgenic VAR1 and VAR2 exhibited microfissures and subsequent dehydration. The other variants, as well as the control MP-1 fruit, did not show any *cwp*^*h*^-related phenotypes, neither microfissures nor dehydration (Fig. 5a).

**Fig. 6:**
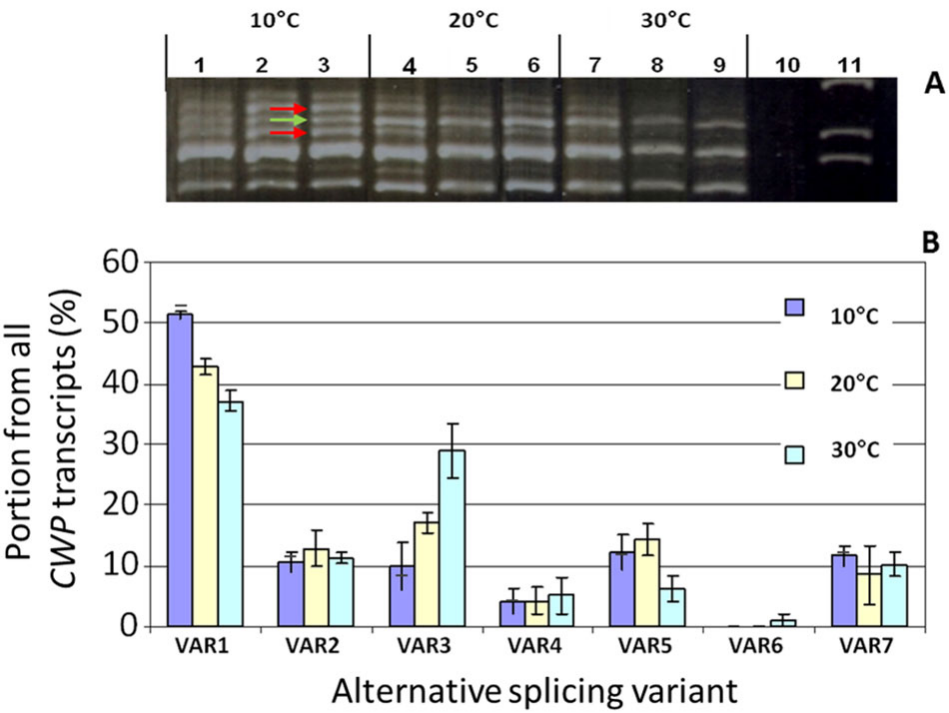
Alternative transcripts in correlation with temperature. **a** Products of PCR reaction performed on *cwp* cDNA after incubation in 10 (lanes 1–3), 20 (lanes 4–6), and 30 °C (lanes 7–9). Lane 10 is a non-template control, lane 11 is a size marker. The arrows point to PCR products significantly affected by temperature. **b** The relative ratio of the alternative transcripts after incubation in the three temperatures

Further analysis of the functional transgenic VAR2 showed that VAR2 itself undergoes alternative splicing, yielding the VAR1 gene product (Fig. 6). Since VAR2 is produced as a result of intron 2 retention, intron 2 can still be removed from VAR2, either to produce VAR1 or by the alternative 3′ process to produce VAR3. PCR amplification of the cDNA of transgenic VAR2 indeed resulted in three bands, corresponding to the three alternative transcripts VAR1, VAR2, and VAR3 (Fig. 5b, c). Thus, we conclude that the longest ORF leading to the 270-amino acid CWP protein is the functional protein.

### Low temperature changes the pattern of alternative splicing leading to a further increase in the functional VAR1

In order to determine whether low temperature also impacts on the pattern of alternative splicing of *cwp*^*h*^, we quantified the different transcript variants in response to temperature treatment of detached IG fruitlets (10, 20, or 30 °C). The alternative transcripts of *cwp*^*h*^ were quantified following PCR cloning of the fruit cDNA and determining transcript size from each treatment in 100 transformed *E. coli* colonies. The results indicated a significant shift in the ratio between two alternative transcripts; low temperature increased the relative proportion of the functional VAR1 transcripts, at the expense of the nonfunctional VAR3 (Fig. 6). Under high temperature, VAR1 accounted for 38% of the transcripts, and this increased to more than 50% under low temperature. VAR3 made up 29% of the transcripts in higher temperature but only 10% in the lower temperature. The remaining variants were present at lower levels and were largely unaffected by temperature. An interesting observation is the increase in VAR5 under low temperature, analogous to the response of VAR1. VAR1 and VAR5 share the alternative splice site, suggesting that this splice site may be specifically affected by temperature.

### Global gene alternative splicing is modified by low temperature

**Fig. 7:**
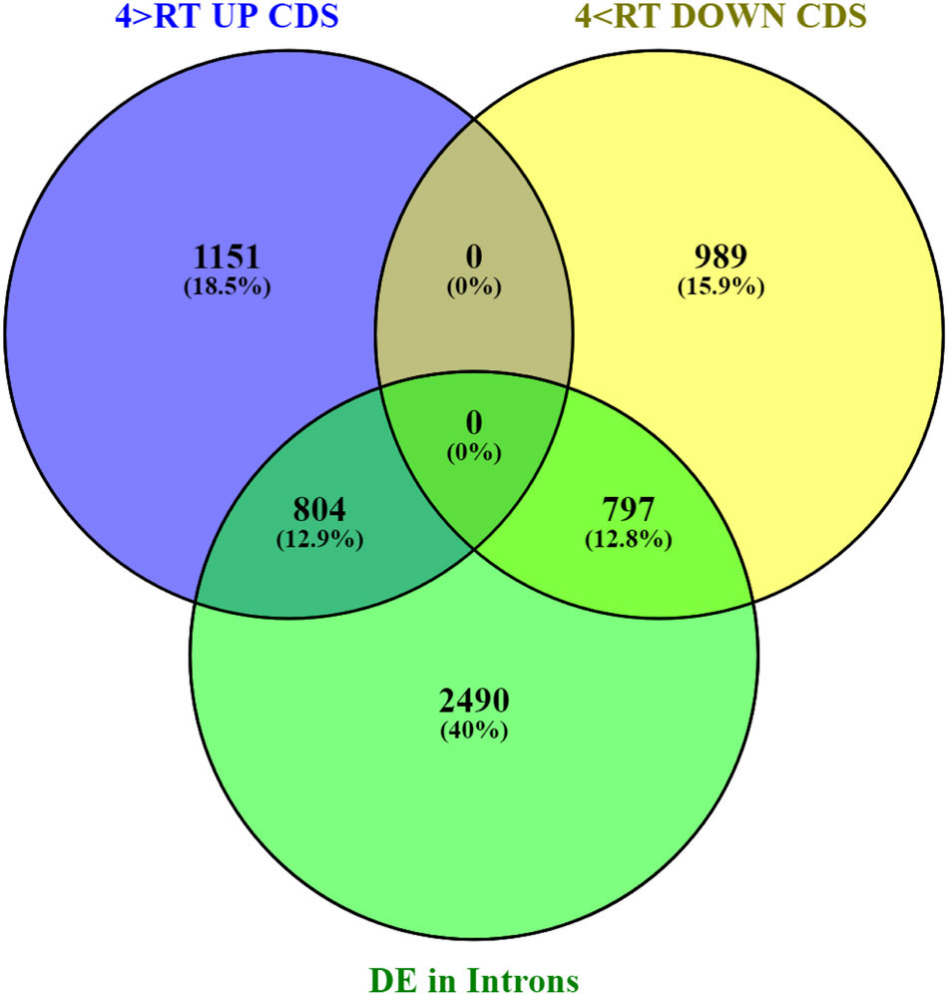
Differential gene expression of coding sequences and of introns in response to low- temperature treatment. The lower circle represents the genes with differential expression of intronic sequences, while the two upper circles indicate the number of differentially upregulated (left) and downregulated (right) genes

Just as *cwp* is not the only gene differentially expressed due to temperature, so too it is not unique in showing alternative splicing in response to temperature. Following the mapping of the RNAseq reads and subsequent FPKM analysis based on the annotated coding sequences of each gene, the reads were similarly mapped specifically to the intron regions of the annotated tomato genome. Thus, we could determine whether temperature affected the expression of intronic regions, serving as an indication of alternative splicing. For the *cwp*^*h*^ genotype, we observed that 4091 genes showed differential expression of intronic regions (>2-fold, padj <0.05) between the two temperature treatments. These data include any significant change in the number of intron-mapped reads per gene. Of the 3741 genes differentially expressed in the *cwp*^*h*^ genotype in response to temperature (Fig. 3), 1601 were also characterized by differential expression of intron regions (Fig. 7). An equal number, about 800, of both low-temperature upregulated and downregulated genes showed differential intron retention.

### Splicing-related genes are differentially expressed under the influence of temperature

**Fig. 8:**
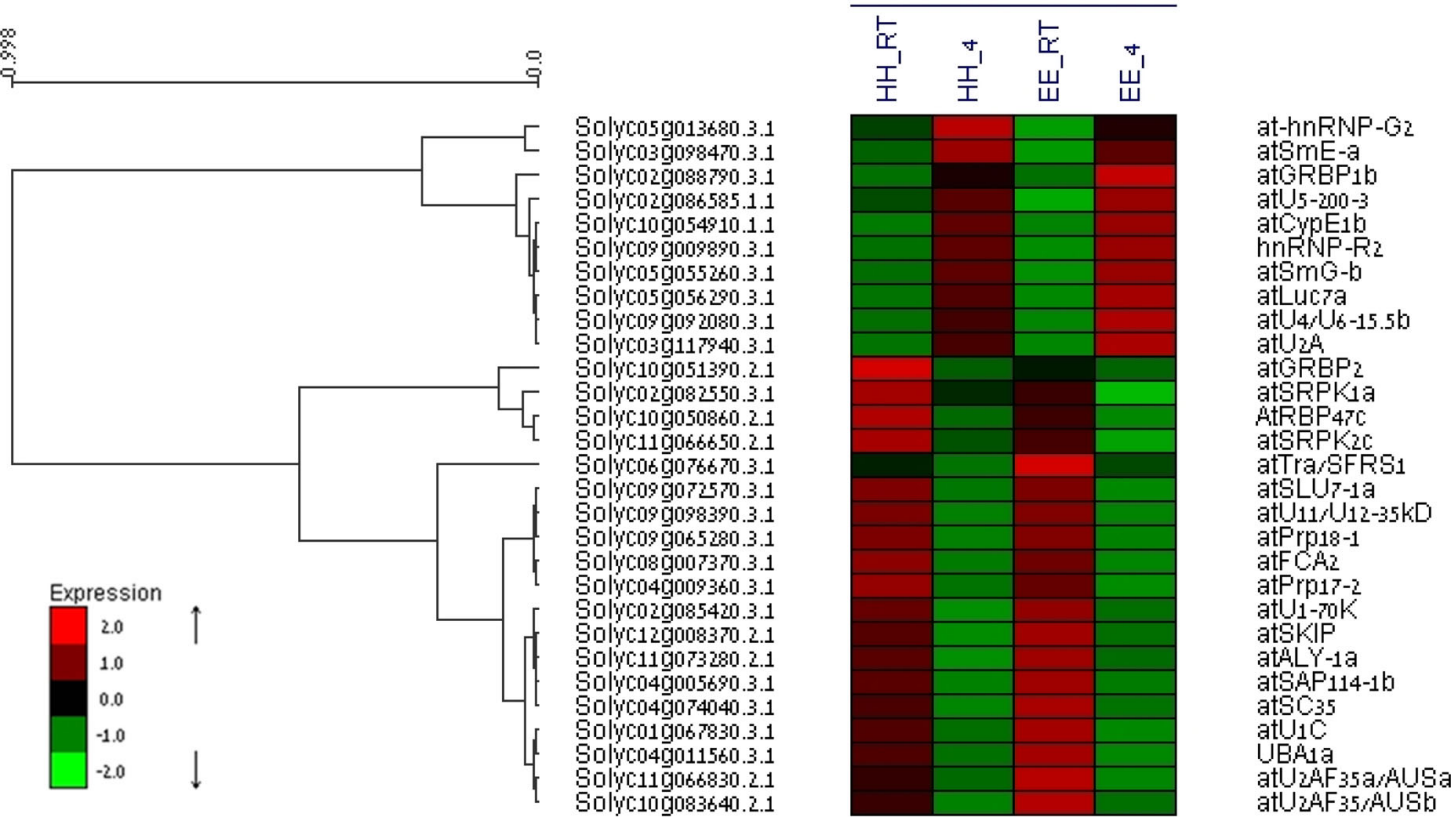
Differential expression of tomato splicing-related genes. Heat map of the 29 splicing-related genes differentially expressed (>2-fold, padj <0.05) in response to temperature. List of the tomato homologs of the Arabidopsis splicing-related genes are listed in Supplementary Excel file 2. Fruitlets of the two genotypes (*cwp*^*h*^, HH; *cwp*^*e*^, EE) were subjected to either 4 or 20 °C for 10 h

In order to shed light on the cause of the temperature-dependent differential alternative splicing, we analyzed the expression data for the known splicing-related genes in the tomato genome. The tomato homologs of the Arabidopsis splicing-related genes were identified by BLAST against the Arabidopsis database (SRGD, http://www.plantgdb.org/SRGD/WB04.php, Wang and Brendel^50^), and 174 tomato genes that showed at least 60% identity to one of the Arabidopsis genes were selected (Supplementary Excel file 2). Analysis of the expression data for these 174 genes indicated that 29 splicing-related genes were differentially expressed (>2-fold, padj <0.05) in response to the low temperature in both allelic genotypes (Fig. 8, Supplementary Excel file 3). Ten genes were upregulated by the low temperature and 19 were downregulated irrespective of the *cwp* genotype.

## Discussion

The wild species *S. habrochaites* allele *cwp*^*h*^ introgressed into the cultivated *S. lycopersicon* leads to the development of microfissures in the tomato fruit cuticle, leading to its subsequent dehydration^3^. In contrast, the cultivated tomato is relatively impervious to water loss, due to a continuous fruit cuticle. Accordingly, the evolutionary silencing of *cwp* expression during *Solanum* evolution is one of the factors that contributed to the evolution under domestication of the fleshy tomato fruit. Our study accounts for the increased severity of microfissuring observed under low temperatures. Both transcriptional and post-transcriptional components of *cwp* gene expression were found to be affected by low temperature.

The relationship between the expression of functional splice variants of the *cwp* gene and the phenotype of microfissures was tested under a range of temperature conditions. A strong correlation was demonstrated between the temperature-modulated *cwp* expression and the intensity of cuticular microfissure, with low temperature causing upregulation of *cwp* expression and severe microfissures, while high temperature leads to reduced *cwp* expression and mild microfissures. In addition, there is a further increase in the relative proportion of the VAR1 splice variant, under low temperatures, and we show that it is the functional variant, thereby compounding the effect of low temperature on the severity of the microfissures.

While the expression of the *cwp*^*h*^ allele is strongly upregulated by low temperature, the *cwp*^*e*^ allele remains silent. Interestingly, the *cwp* gene is the only such gene that is silent in the *cwp*^*e*^ genotype, irrespective of temperature, and upregulated significantly in the *cwp*^*h*^ genotype in response to low temperature.

Ambient temperature is a well-established factor controlling gene expression in plants. Our results indicate that in developing tomato fruit, low temperature of 4 °C for 10 h is perceived by the fruit tissue per se and leads to the differential expression of ~ 6000 of the expressed genes, or ca. 25% of the tomato genome. This is in general agreement with other studies, including those of tomato tissue. For example, Chen et al.^25^ similarly reported about 6000 genes differentially expressed in tomato leaf tissue following 12 h at 4 °C.

Alternative splicing plays an important role in adaptation of plants to abiotic stress and environmental constraints^22,52,53^. Evidence for the effect of temperature stresses on the plant transcriptome was shown by Filichkin et al.^36^, who observed not only an altered abundance of many transcripts but also changes in their alternative splicing pattern. More recently, this area of study has benefitted from HTS in showing the temperature effects on alternative splicing patterns, including in tomato^25–28^. While the effect of low temperature on alternative splicing patterns is a global transcriptome phenomenon, the physiological impact is likely due to individual gene transcript modifications. One of the earliest reports showing the effect of low temperature on a physiological process via alternative splicing of a particular gene was the study of the low-temperature effect on a potato invertase gene related to cold-sweetening during potato storage^54^. In this study, temperature impacted on the retention of the smallest known plant exon, 9 bp, which translates to the NDP catalytic domain, with concomitant implications on sucrose hydrolysis.

Our results with respect to the occurrence of alternative splicing are also in general agreement with other studies. Particularly regarding young tomato fruit, Sun and Xiao^27^ reported that AS characterizes 60% of multi-exon genes during young tomato fruit development, similar to our results. Since our RNAseq reads were single-end 50 bp (SE50), the ability to fully characterize alternative splicing was hampered. Therefore, our observation was based on the differential expression of introns and is not a strict analysis of alternative splicing. Nevertheless, our results clearly indicate that *cwp* is not the only differentially expressed gene that also undergoes alternative splicing. Not all AS genes affected by temperature are also differentially expressed, and in 2490 genes temperature affects only the variant splicing mechanism. Reciprocally, 2140 genes are differentially expressed but do not show differential intron expression. *cwp* is one of about 1600 genes that are both differentially expressed and alternatively spliced in response to low temperature, However, in the case of *cwp*, this leads to a synergistic effect on the production of functional processed mRNA and hence the horticultural phenotype.

The effect of temperature on alternative splicing is an established phenomenon^30,31,55^, and the response to temperature is rapid^56^, likely occurring in even less than the 10-h treatment we report. Significantly, the splicing mechanism itself is affected by temperature. Verhage et al.^57^ recently reported that even under modest temperature changes, there were significant effects on splicing-related genes, suggesting that the whole spliceosome is sensitive to temperature. They present a two-step model of temperature control of plant development: first, the alternative splicing of splicing-mechanism genes, followed by the subsequent alternative splicing of downstream genes, including genes of the transcriptional network^25,55^. To this, we might add a further initial step to the model based on our results: the differential regulation of gene expression of the splicing-mechanism genes, impacting on the reservoir for alternative splicing variants.

The important contribution of the endogenous splicing machinery is emphasized particularly in the introgression lines. While the endogenous *cwp*^*h*^ allele in *S. habrochaites* fruit showed three alternative transcripts, the *cwp*^*h*^ introgressed in the *S. lycopersicon* background that expressed seven variants. This suggests a genetic background effect for the alternative splicing process, based perhaps on genetic variability between the species in splicing-mechanism genes, either due to genetic variability in their biochemical function or to differences in expression or its own alternative splicing.

We show that the functional variant with respect to microfissures is the VAR1, with the longest mRNA product. The other variants are characterized by premature stop codons that lead to shortened reading frames and truncated proteins. These RNAs may likely be targeted to rapid degradation through the nonsense-mediated-decay (NMD) pathway^58,59^. As such, the case of *cwp* is an example of alternative splicing exhibiting physiological significance^32^. Nevertheless, we cannot conclude with certainty that in fact the VAR1 is the “functional” variant since we do not yet know what the physiological function of *cwp* actually is. While *cwp* is expressed in the green-fruited wild species of tomato^3^, these wild species fruit do not exhibit microfissures^7^, indicating that the physiological role of *cwp* is not in developing microfissures per se. Rather, it is only in the background of the cultivated tomato that the phenotype of microfissures is observed when *cwp* is expressed. One could hypothesize that cuticular microfissuring is an unrelated phenotype produced serendipitously by the interaction of *cwp* with *S. lycopersicon* components of fruit rind development, and that the physiological role of *cwp* in the primitive wild species and other *Solanaceae* is to be found elsewhere. Thus, it is still possible that the other alternative transcripts do indeed have a function, perhaps even the natural physiological one^60^. The unknown function of *cwp* remains to be revealed, and the effect of low temperature on upregulating its expression may be useful in this pursuit.

## Supplementary information

Supplementary file- sequences

Supplementary Figures and Tables

Supplemental Excel File 1

Supplemental Excel File 2

Supplemental Excel File 3
