# Supplementary material for: Low temperature upregulates cwp expression and modifies alternative splicing patterns, increasing the severity of cwp-induced tomato fruit cuticular microfissures
Source: Hortic Res. 2019 Nov 8;6:122. doi: 10.1038/s41438-019-0204-9 (PMC6838111; doi:10.1038/s41438-019-0204-9)
Supplement: Supplementary file 1 — Supplementary file- sequences [file 41438_2019_204_MOESM1_ESM.docx]

**Supplementary word file 1**

The sequence of the genomic DNA of *CWP^H^* allele and the sequences of the seven alternative transcripts (named VAR1 to VAR7)

*>CWP* genomic sequence

ATGTGTATAGTAGTGTTTATTTGGGAAGCAGATAGTAGATATTCATTAGTGTTATTATTGAATAGAGATGAATATCATAATAGGCCAACAAAGGAAGTTCATTGGTGGGAAGATGGAGAAATTGTTGGTGGCAAAGATGAAGTTGGTGGTGGCACTTGGTTGGCTTCTTCAACTAATGGTAAATTGGCTTTTCTTACTAATGTTTTGGAAATTCATACACTTCCTCATGCCAAAACTAGAGGTGATCTACCCGTTCGATTTTTACAGGTACGATTAAATTCTTTATATATTATACGTTAATATGTTTGATCTTTCATTTTGGTTTTGTTATACGAAGGACGAGACCTAGAGGTCTTTAAGACAAAACATAAATATGCATCATAGTCATAAACTTTCAATAAATATTCAATTTTGAATATGCGCTTTCAAAGGTATTACAAGTTGAGTACTAAAGGAATTGAGTTTATCAAGATTAAATTTTGAATTTGATTCTTTTGATCATGATTAATAGTAATGTTAAATCTTGTCCTTATTGGAGTATATATATGATCAATAAATCAAGATTTTAAATTGTAGTATAATCTTAATTTTAAAGAATATTAATGTTGTAAAATTTTAGATTTAACAAACACAAAAATCATATTTGTATGTTATAACTATAGTTTGTATAGTTGCGCTCAATATGTTTGTTCGCGAGCTGTTAATATGTCACTATTTCGGTTTACATATACAAAAGAGATCAATTGCATAATTTTGTTTNGCATATACNTNTTAAACATGATACATAATAGAAATTTCATTNATTGTGTAATATATCTTTGTATAAAGCAAGAAAGAGCGAAACACAACAGAAAACTGGATAGGGAAATATTTATATTTTGTATAGTTATAAGTGTATATGACGGAAATATACGTAATTATTTTTTATACATGATTTTCTCTCGCTTTTATGCAAACACAAACACAATTTATACATTTGTTTTTGTGTAAAGTGAGAGTGGCGAGCGAGATTCTATAGAGAGAGAACCAAATGAAAATATATGTATTATATGCAGTTTTCTGTAGTTTTATACAAATACAAACACAATTTATACATTTATTTTTGTGTATGAGAGAGGCGAGTGAGATTCTCNGGGGAGGAAAATATATGTATATATACAGTTTTGTTTCGCTATAAACAAACAGAACACATTTTATACATTTGTATTTGTATAAAACAAGAGAGACGAGGGAGAAACTGCTCAACGAGAAATTCAGGAAGAGAGGTGAATGACAACTATTTGTTACGAGTTGCAAGTAAATCAAACTGCGACTATAACATTTAGTTTGAATTAATAATTTGTTATTTTAAACGATTTTCCGTAAAATTTAATTGTTAATTGCAGAGCAATAAAAGCCCAATGGAGTTTGCAAAAGGGCTGGTGAATGAAGGGAATGAATACAATGGGTTTAATTTAATTTTGGCAGATATTGAAACTAAAAAAATGGTATATGTAACAAATAGGCCCAAAGGAGAGCCCATAACAATACAAGAAGTCCAACCAGGTATTCATGTGCTGTCCAATGCAAAACTGGACTCTCCTTGGCCCAAGGTAAGAATTCTAATGGGCTTTTTTCGATCGATATACATAAATTATACAAATGATATGCTTTTGGTTGTTCATTTCAGGCTCAAAGACTGAAGTTAAATTTTAAGAAAATGATGGATGTTTACGAAGTGAATGACGAGAAAATCTGCGTCAAAGATATGATAGAAAAATTGATGAGAGATACCACTAAAGCTGATAAAAGTAAATTGCCTTGTATTTGTTCTACGGACTGGGAGTTGGAACTTAGCTCTATTTTCGTGGAAGTTGACACTgCACTGGTAATTCATACCGCGTTATAACTAATATGTTTGTTTGATTTTAACGTACTCAAACGATGATAAAGGTTAAAGTAGATATACAAACATTTTAAAAATAATTGAAATAGTTCAATAATAGAAGTGTACATATCATTAACATAGTTTGATGGGTTTTTTTGGTGGTGTGAATATGTAGGGGTGTTATGGTACTAGAAGTACAACAGCATTGACAATTGAAGTGGGAGGAGAAGTAAGCTTTTATGAGTTGTACCTTGAGAACAACATGTGGAAAGAGCAAATTGTCAACTATCGGATTGAAAAACTCCAAATGCAATAA

>VAR1

ATGTGTATAGTAGTGTTTATTTGGGAAGCAGATAGTAGATATTCATTAGTGTTATTATTGAATAGAGATGAATATCATAATAGGCCAACAAAGGAAGTTCATTGGTGGGAAGATGGAGAAATTGTTGGTGGCAAAGATGAAGTTGGTGGTGGCACTTGGTTGGCTTCTTCAACTAATGGTAAATTGGCTTTTCTTACTAATGTTTTGGAACTTCATACACTTCCTCATGCCAAAACTAGAGGTGACCTACCTCTTCGATTTTTACAGAGCAATAAAAGCCCAATGGAGTTTGCAAAAGAGTTGGTGAATGAAGGGAATGAATACAATGGGTTTAATTTAATTTTGGCAGATATTGAAACTAAAAAAATGGTATATGTAACAAATAGGCCCAAAGGAGAGCCCATAACAATACAAGAAGTCCAACCAGGTATTCATGTGCTGTCCAATGCAAAACTGGACTCTCCTTGGCCCAAGGCTCAAAGACTGAAGTTAAATTTTAAGAAAATGATGGATGTTTACGAAGTGAATGACGAGAAAATCTGCGTCAAAGATATGATAGAAAAATTGATGAGAGATACCACTAAAGCTGATAAAAGTAAATTGCCTTGTATTTGTTCTACAGACTGGGAGTTGGAACTTAGCTCTATTTTCGTGGAAGTTGACACTGCACTGGGGTGTTATGGTACTAGAAGTACAACAGCATTGACAATTGAAGTGGGAGGAGAAGTAAGCTTTTATGAGTTGTACCTTGAGAACAACATGTGGAAAGAGCAAATTGTCAACTATCGGATTGAAAAACTCCAAATGCAATAA

>VAR2

ATGTGTATAGTAGTGTTTATTTGGGAAGCAGATAGTAGATATTCATTAGTGTTATTATTGAATAGAGATGAATATCATAATAGGCCAACAAAGGAAGTTCATTGGTGGGAAGATGGAGAAATTGTTGGTGGCAAAGATGAAGTTGGTGGTGGCACTTGGTTGGCTTCTTCAACTAATGGTAAATTGGCTTTCTTACTAATGTTTTGGAAATTCATACACTTCCTCATGCCAAAACTAGAGGTGATCTACCCGTTCGATTTTTACAGAGCAATAAAAGCCCAATGGAGTTTGCAAAAGAGTTGGTGAATGAAGGGAATGAATACAATGGGTTTAATTTAATTTTGGCAGATATTGAAACTAAAAAAATGGTATATGTAACAAATAGGCCCAAAGGAGAGCCCATAACAATACAAGAAGTCCAACCAGGTATTCATGTGCTGTCCAATGCAAAACTGGACTCTCCTTGGCCCAAGGTAAGAATTCTAATGGGCTTTTTTCGATCGATATACATAAATTATACAAATGATATGCTTTTGGTTGTTCATTTCAGGCTCAAAGACTGAAGTTAAATTTTAAGAAAATGATGGATGTTTACGAAGTGAATGACGAGAAAATCTGCGTCAAAGATATGATAGAAAAATTGATGAGAGATACCACTAAAGCTGATAAAAGTAAATTGCCTTGTATTTGTTCTACAGACTGGGAGTTGGAACTTAGCTCTATTTTCGTGGAAGTTGACACTGCACTGGGGTGTTATGGTACTAGAAGTACAACAGCATTGACAATTGAAGTGGGAGGAGAAGTAAGCTTTTATGAGTTGTACCTTGAGAACAACATGTGGAAAGAGCAAATTGTCAACTATCGGATTGAAAAACTCCAAATGCAATAA

>VAR3

ATGTGTATAGTAGTGTTTATTTGGGAAGCAGATAGTAGATATTCATTAGTGTTATTATTGAATAGAGATGAATATCATAATAGGCCAACAAAGGAAGTTCATTGGTGGGAAGATGGAGAAATTGTTGGTGGCAAAGATGAAGTTGGTGGTGGCACTTGGTTGGCTTCTTCAACTAATGGTAAATTGGCTTTTCTTACTAATGTTTTGGAACTTCATACACTTCCTCATGCCAAAACTAGAGGTGACCTACCTCTTCGATTTTTACAGAGCAATAAAAGCCCAATGGAGTTTGCAAAAGAGTTGGTGAATGAAGGGAATGAATACAATGGGTTTAATTTAATTTTGGCAGATATTGAAACTAAAAAAATGGTATATGTAACAAATAGGCCCAAAGGAGAGCCCATAACAATACAAGAAGTCCAACCAGGTATTCATGTGCTGTCCAATGCAAAACTGGACTCTCCTTGGCCCAAGACTGGGAGTTGGAACTTAGCTCTATTTTCGTGGAAGTTGACACTgCACTGGGGTGTTATGGTACTAGAAGTACAACAGCATTGACAATTGAAGTGGGAGGAGAAGTAAGCTTTTATGAGTTGTACCTTGAGAACAACATGTGGAAAGAGCAAATTGTCAACTATCGGATTGAAAAACTCCAAATGCAATAA

>VAR4

ATGTGTATAGTAGTGTTTATTTGGGAAGCAGATAGTAGATATTCATTAGTGTTATTATTGAATAGAGATGAATATCATAATAGGCCAACAAAGGAAGTTCATTGGTGGGAAGATGGAGAAATTGTTGGTGGCAAAGATGAAGTTGGTGGTGGCACTTGGTTGGCTTCTTCAACTAATGAGCAATAAAAGCCCAATGGAGTTTGCAAAAGAGTTGGTGAATGAAGGGAATGAATACAATGGGTTTAATTTAATTTTGGCAGATATTGAAACTAAAAAAATGGTATATGTAACAAATAGGCCCAAAGGAGAGCCCATAACAATACAAGAAGTCCAACCAGGTATTCATGTGCTGTCCAATGCAAAACTGGACTCTCCTTGGCCCAAGACTGGGAGTTGGAACTTAGCTCTATTTTCGTGGAAGTTGACACTGCACTGGGGTGTTATGGTACTAGAAGTACAACAGCATTGACAATTGAAGTGGGAGGAGAAGTAAGCTTTTATGAGTTGTACCTTGAGAACAACATGTGGAAAGAGCAAATTGTCAACTATCGGATTGAAAAACTCCAAATGCAATAA

>VAR5

ATGTGTATAGTAGTGTTTATTTGGGAAGCAGATAGTAGATATTCATTAGTGTTATTATTGAATAGAGATGAATATCATAATAGGCCAACAAAGGAAGTTCATTGGTGGGAAGATGGAGAAATTGTTGGTGGCAAAGATGAAGTTGGTGGTGGCACTTGGTTGGCTTCTTCAACTAATGAGCAATAAAAGCCCAATGGAGTTTGCAAAAGAGTTGGTGAATGAAGGGAATGAATACAATGGGTTTAATTTAATTTTGGCAGATATTGAAACTAAAAAAATGGTATATGTAACAAATAGGCCCAAAGGAGAGCCCATAACAATACAAGAAGTCCAACCAGGTATTCATGTGCTGTCCAATGCAAAACTGGACTCTCCTTGGCCCAAGGCTCAAAGACTGAAGTTAAATTTTAAGAAAATGATGGATGTTTACGAAGTGAATGACGAGAAAATCTGCGTCAAAGATATGATAGAAAAATTGATGAGAGATACCACTAAAGCTGATAAAAGTAAATTGCCTTGTATTTGTTCTACAGACTGGGAGTTGGAACTTAGCTCTATTTTCGTGGAAGTTGACACTGCACTGGGGTGTTATGGTACTAGAAGTACAACAGCATTGACAATTGAAGTGGGAGGAGAAGTAAGCTTTTATGAGTTGTACCTTGAGAACAACATGTGGAAAGAGCAAATTGTCAACTATCGGATTGAAAAACTCCAAATGCAATAA

>VAR6

ATGTGTATAGTAGTGTTTATTTGGGAAGCAGATAGTAGATATTCATTAGTGTTATTATTGAATAGAGATGAATATCATAATAGGCCAACAAAGGAAGTTCATTGGTGGGAAGATGGAGAAATTGTTGGTGGCAAAGATGAAGTTGGTGGTGGCACTTGGTTGGCTTCTTCAACTAATGACTGGGAGTTGGAACTTAGCTCTATTTTCGTGGAAGTTGACACTGCACTGGGGTGTTATGGTACTAGAAGTACAACAGCATTGACAATTGAAGTGGGAGGAGAAGTAAGCTTTTATGAGTTGTACCTTGAGAACAACATGTGGAAAGAGCAAATTGTCAACTATCGGATTGAAAAACTCCAAATGCAATAA

>VAR7

ATGTGTATAGTAGTGTTTATTTGGGAAGCAGATAGTAGATATTCATTAGTGTTATTATTGAATAGAGATGAATATCATAATAGGCCAACAAAGGAAGTTCATTGGTGGGAAGATGGAGAAATTGTTGGTGGCAAAGATGAAGTTGGTGGTGGCACTTGGTTGGCTTCTTCAACTAATGAGCAATAAAAGCCCAATGGAGTTTGCAAAAGAGTTGGTGAATGAAGGGAATGAATACAATGGGTTTAATTTAATTTTGGCAGATATTGAAACTAAAAAAATGGTATATGTAACAAATAGGCCCAAAGGAGAGCCCATAACAATACAAGAAGTCCAACCAGGTATTCATGTGCTGTCCAATGCAAAACTGGACTCTCCTTGGCCCAAGGTAAGACTTCTAATGGGCTTTTTTCGATCGATATACGTAAATTATACACATGATATGCTTTTGGTTGTTAATTTCAGGCTCAAAGACTGAAGTTAAATTTTAAGAAAATGATGGATGTTTACGAAGTGAATGACGAGAAAATCTGCGTCAAAGATATGATAGAAAAATTGATGAGAGATACCACTAAAGCTGATAAAAGTAAATTGCCTTGTATTTGTTCTACAGACTGGGAGTTGGAACTTAGCTCTATTTTCGTGGAAGTTGACACTGCACTGGGGTGTTATGGTACTAGAAGTACAACAGCATTGACAATTGAAGTGGGAGGAGAAGTAAGCTTTTATGAGTTGTACCTTGAGAACAACATGTGGAAAGAGCAAATTGTCAACTATCGGATTGAAAAACTCCAAATGCAATAA
