# Supplementary material for: Low temperature upregulates cwp expression and modifies alternative splicing patterns, increasing the severity of cwp-induced tomato fruit cuticular microfissures
Source: Hortic Res. 2019 Nov 8;6:122. doi: 10.1038/s41438-019-0204-9 (PMC6838111; doi:10.1038/s41438-019-0204-9)

## Slide 1
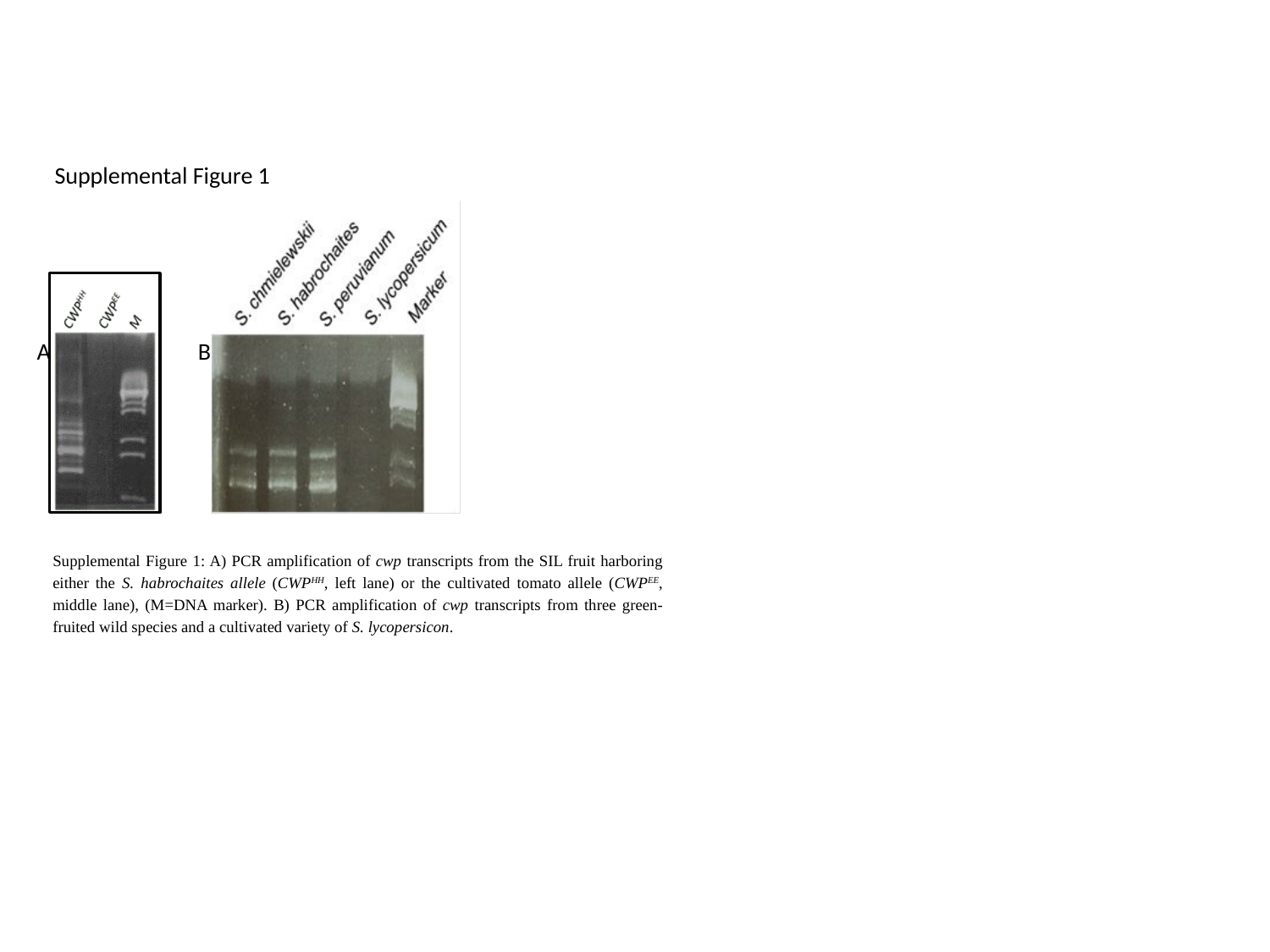

Supplemental Figure 1
A
B
Supplemental Figure 1: A) PCR amplification of cwp transcripts from the SIL fruit harboring either the S. habrochaites allele (CWPHH, left lane) or the cultivated tomato allele (CWPEE, middle lane), (M=DNA marker). B) PCR amplification of cwp transcripts from three green-fruited wild species and a cultivated variety of S. lycopersicon.

## Slide 2
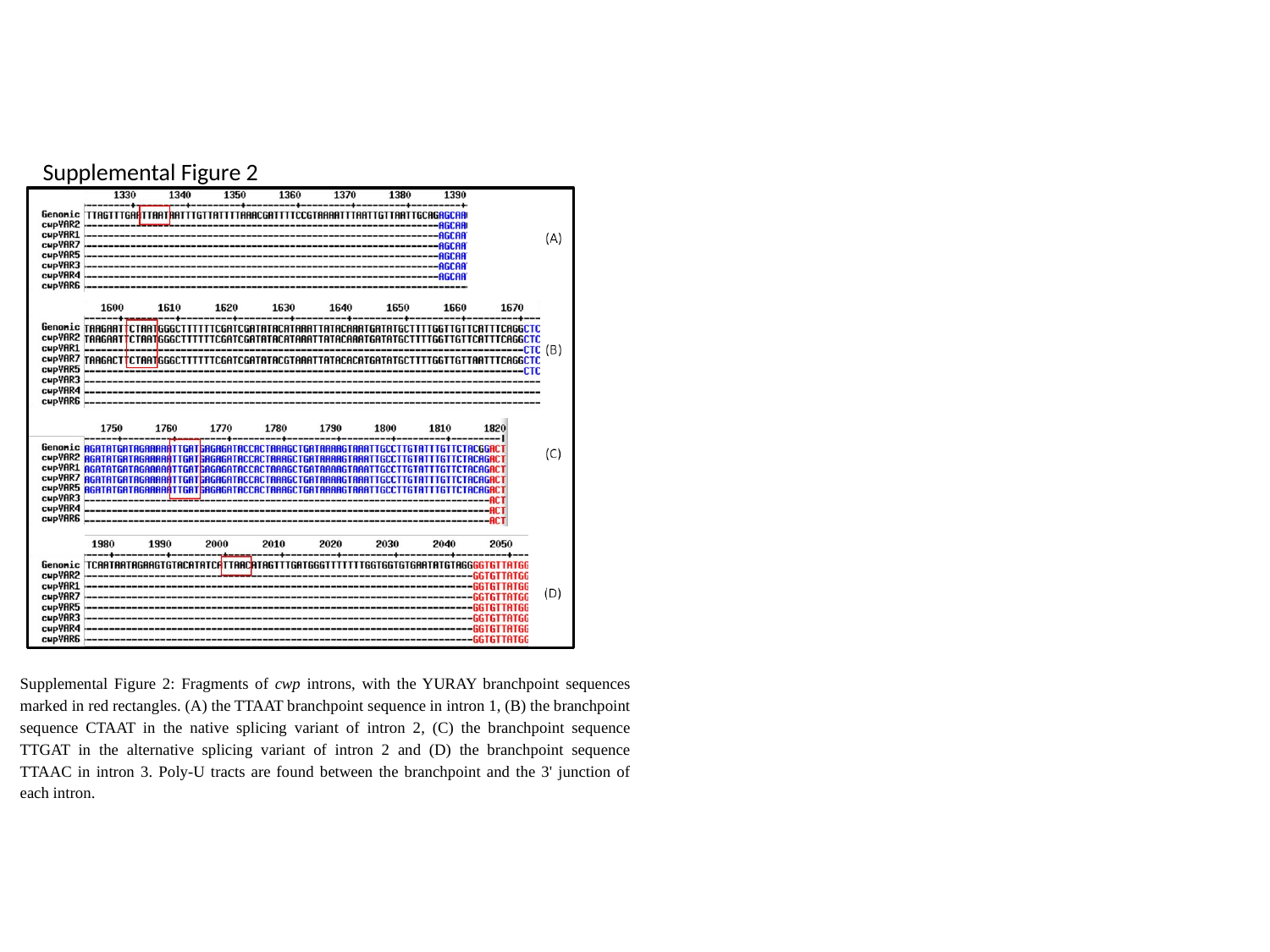

Supplemental Figure 2
Supplemental Figure 2: Fragments of cwp introns, with the YURAY branchpoint sequences marked in red rectangles. (A) the TTAAT branchpoint sequence in intron 1, (B) the branchpoint sequence CTAAT in the native splicing variant of intron 2, (C) the branchpoint sequence TTGAT in the alternative splicing variant of intron 2 and (D) the branchpoint sequence TTAAC in intron 3. Poly-U tracts are found between the branchpoint and the 3' junction of each intron.

## Slide 3
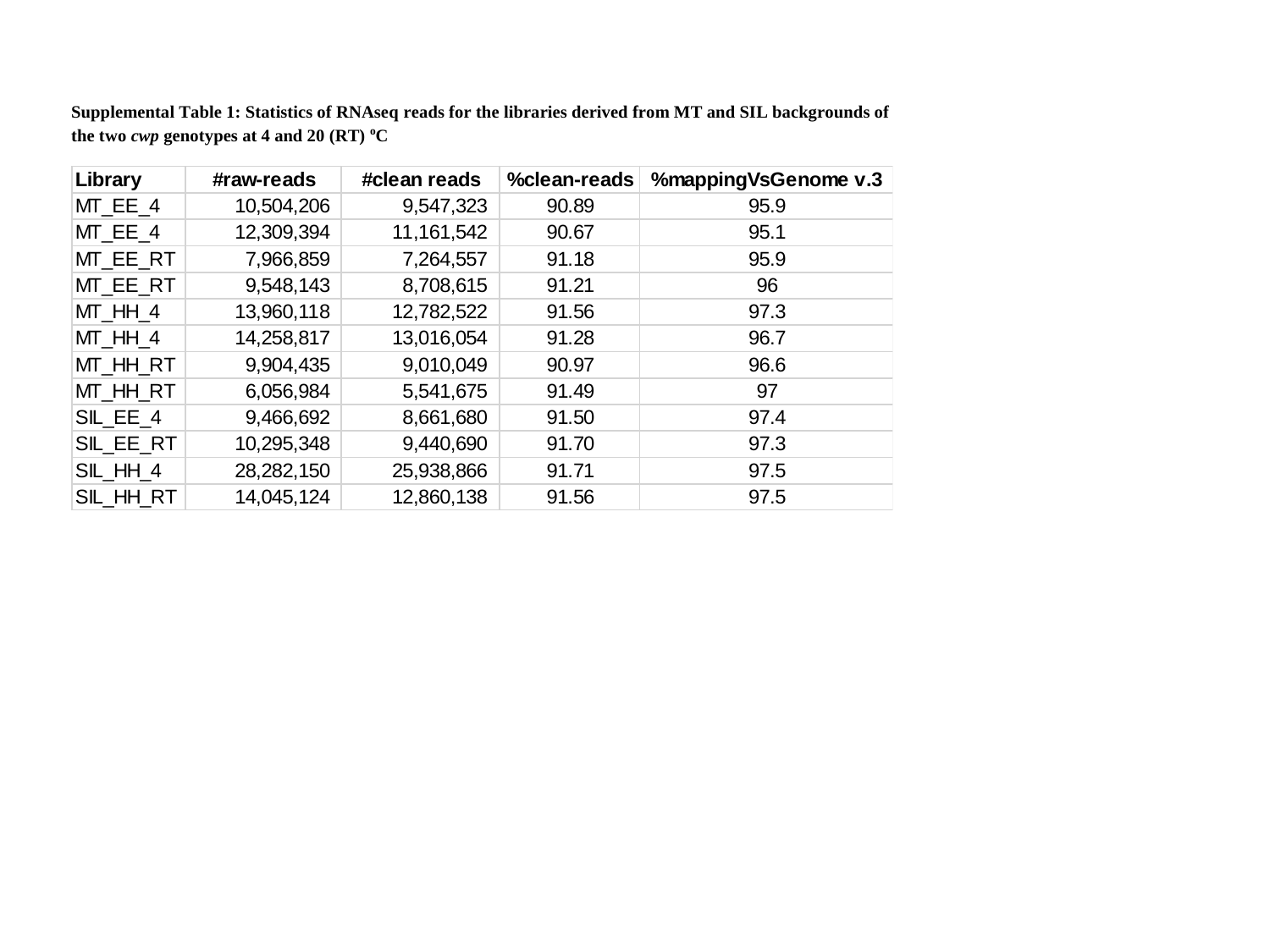

## Slide 4
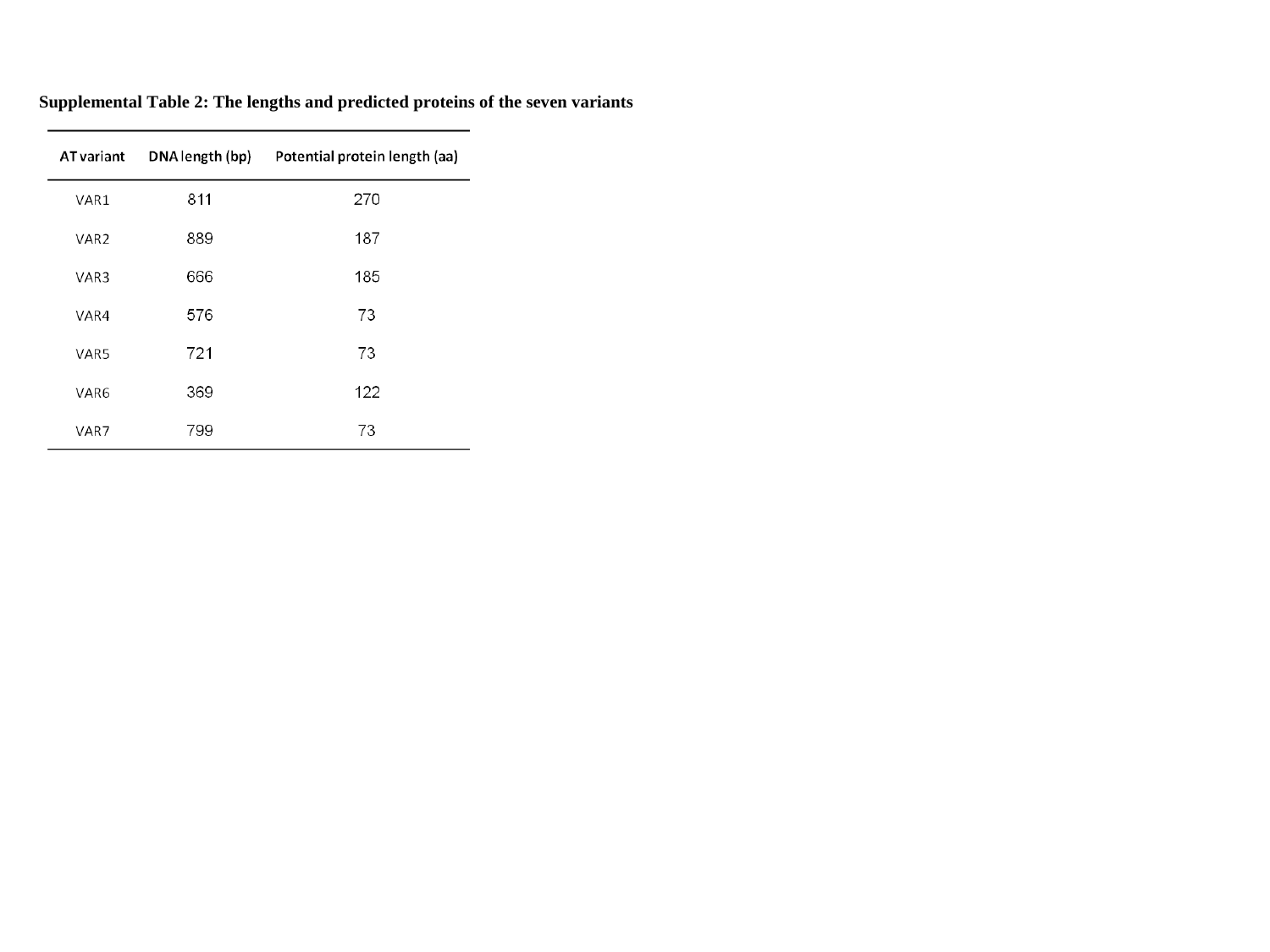

Supplement: Supplementary file 2 — Supplementary Figures and Tables [file 41438_2019_204_MOESM2_ESM.pptx]
